# Hypercapnia impaired cognitive and memory functions in obese patients with obstructive sleep apnoea

**DOI:** 10.1038/s41598-018-35797-3

**Published:** 2018-12-03

**Authors:** Shu-Chin Kung, Yu-Chih Shen, En-Ting Chang, Ya-Ling Hong, Ling-Yi Wang

**Affiliations:** 1 Department of Human Development and Psychology, Tzu Chi University, Hualien, Taiwan; 2 Department of Psychiatry, Buddhist Tzu Chi General Hospital, Hualien, Taiwan; 3 School of Medicine, Tzu Chi University, Hualien, Taiwan; 4 Department of Chest Medicine, Buddhist Tzu Chi General Hospital, Hualien, Taiwan; 5 Epidemiology and Biostatistics Consulting Center, Buddhist Tzu Chi General Hospital, Hualien, Taiwan

## Abstract

Obstructive sleep apnoea (OSA) is a sleep disorder involving repeated nocturnal desaturation and sleep fragmentation. OSA can result in decreased daytime alertness and neurocognitive dysfunction. Hypercapnia status is also related to neurocognitive dysfunction in patients with pulmonary diseases. We evaluated the effects of hypercapnia on cognitive performance and memory function in a prospective case-controlled study. We enrolled thirty-nine obese patients with OSA and collected their arterial blood samples. All the participants provided arterial blood samples, and completed two questionnaires (the Pittsburgh Sleep Quality Index and the Epworth Sleepiness Scale) and six cognitive tasks (the psychomotor vigilance task [PVT], the Stroop task, the Eriksen flanker task, processing speed [DSST], and verbal and visual memory [LM&FM]), which were used to evaluate daytime sleepiness, cognitive function, and memory function within one week of a polysomnographic study. When compared to the OSA without diurnal hypoventilation, the patients with stable hypercapnia (OHS) had increased reaction times in the PVT, Stroop task, and flanker task. Hypercapnic obese patients with OSA also had comparatively significantly lower scores in the processing speed and logical memory tests. OHS had increased reaction times in the attention and cognitive function assessments, and deficits in the logical memory, when compared to those with OSA without diurnal hypoventilation.

## Introduction

Obstructive sleep apnoea (OSA) syndrome involves recurrent upper airway collapses, leading to hypoxemia and sleep fragmentation during sleep^1^. The clinical features of OSA include snoring, daytime sleepiness, and sleep fragmentation^2^. OSA were found significant negative effect sizes for all cognitive domains with the exception of the cognitive domain of perception^3^. Many studies reported that the patients with OSA have significant deficits in cognitive function, attention, and memory function^4–6^. Further, in one study, Jackson *et al*. reported significant impairments in attention, vigilance, memory and executive function in the patients with OSA^7,8^. These cognitive impairments can lead to difficulty in concentration, increased forgetfulness, and problems with decision-making.

Memory dysfunction is another neurocognitive impairment observed in the patients with OSA syndrome^9^. The mechanism of the relationship between OSA and memory impairment is not clear. The mechanism of cognitive and memory impairment in OSA might involve the effects of sleep fragmentation and regular intermittent hypoxemia injury the brain cell and the hippocampus^3,10^. In a study by Twigg *et al*., obstructive sleep apnoea was found to be associated with the impairment of the verbal memory, but not that of the visual memory^6^. However, the apnoea/hypopnoea index (AHI) and daytime sleepiness were not correlated with memory impairment.

Although the relations between OSA and cognitive impairment has been well-described in several studies, the effects of hypercapnia on cognitive function are poorly understood. Hypercapnia status has been shown to predict mild cognitive impairment in aging rodents^11^. In addition, patients with OSA and chronic obstructive lung disease (COPD) have been reported to have deficits in attention, psychomotor speed, executive function, language abilities and memory function^12,13^. Previously, Hypoxemia and/or hypercarbia have been hypothesised as the factors underlying neurocognitive dysfunction^13^. However, the influence of hypercapnic status on cognition still needs to be determined.

We aimed to compare the impact of chronic stable hypercapnic status (arterial blood gas pH > 7.35 and partial pressure of CO_2_ [PaCO_2_] > 45 mmHg) on the neurocognitive function (attention, executive function, and memory function) in the patients with OSA. We hypothesized that the hypercapnic would impaired neurocognitive function in OSA patients.

## Methods

In this prospective study, we enrolled consecutive 39 obese patients (body mass index [BMI] ≥ 30) with moderate-to-severe OSA (AHI score ≥ 15, derived using standard polysomnography) from 150 clinical patients at the Sleep Center at the Hualien Tzu-Chi General Hospital during 2016 and 2017. All the enrolled subjects underwent an arterial blood test within 1 week of the neurocognitive test and completed the neurocognitive tests prior to any treatment of their OSA. All the enrolled subjects completed the Epworth Sleepiness Scale (ESS), the Pittsburgh Sleep Quality Index (PSQI), the psychomotor vigilance task, the Eriksen flanker task, the Stroop task, and a memory test. All the enrolled subjects were >20 years in age. Those with BMI (body mass index) ≧30 can met our obesity criteria. The following patients were excluded from the study: those who had previously been diagnosed or treated for any neurological or psychological diseases that would interfered with the neurocognitive tests, those with chronic lung (standard pulmonary function shows obstructive or restrictive lung disease) or neuromuscular diseases that would have interfered with the neurocognitive task, and those who could not cooperate with the tasks. Patients with unstable vital signs (unstable blood pressure, tachypnoea [respiratory rate > 28 bpm], or acidaemia [pH < 7.35]) were also excluded from our study. The hospital’s Institutional Review Board approved this study (IRB105-20-A), and all the participants provided informed consent. All the research methods were performed in accordance with the relevant guidelines and regulations. The study was funded by Buddhist Tzu Chi General Hospital, Hualien, Taiwan (TCRD 106-19).

### Polysomnography

All the patients underwent one night of standard type-1 attended polysomnography (Embla A10, Embla; Broomfield, CO) at our Sleep Center. Sleep and arousal periods were scored according to the standard criteria^2^. AHI and 4% Oxygen Desaturation Index (ODI) were calculated as the markers of disease severity.

### Arterial blood test

All enrolled subjects provided arterial blood samples for testing within 1 week of the neurocognitive test during the daytime. According to the result of arterial blood test, the subjects were divided into PaCO2 ≧ 45 mmHg and PaCO2 < 45 mmHg 2 groups.

### Measures

#### Questionnaires

The ESS is an 8-item questionnaire asking respondents to answer each question using a number between 0 to 3, with a total score ranging from 0 (minimum) to 24 (maximum)^14^. All enrolled subjects also completed the PSQI including 19 sleep item^15^.

#### **Studied tasks**

The enrolled patients were asked to complete attention (psychomotor vigilance task), cognitive (Flanker and Stroop tasks) and memory task. All the cognitive tasks for these tests were generated using E-prime 2.0^16^.

### Attention and processing speed tasks

#### Psychomotor vigilance task

The patients were instructed to press a button as quickly as possible when a red 500-ms counter appeared on a small screen. We assessed and collected the following outcome measures of (1) omission rate (OR); (2) lape rate (LR); (3) hit rate (HR); (4) fast rate (FR); (5) reciprocal reaction time (RT); (6) measure of speed (1/RT); (7) false RT; (8) the fastest 10% of the RT and the slowest 10% of RT^16,17^.

#### Digit Symbol Substitution Test (DSST)

The DSST is a component of the Wechsler Adult Intelligence Scale-Third Edition (WAIS- **III**). The test requires the subjects to transcribe a unique geometric symbol with its corresponding Arabic number. The outcome measures of correct items completed within 120 seconds^16^.

### Executive tasks

#### Flanker task

In this study, we used the computerized arrow version of the Flanker task. The outcome we collected the measurements of: (1) omission rate (OR), (2) error rate (ER), (3) accuracy rate (AR); (4) correct RT^16,18^.

#### Colour-word Stroop task

Subjects were presented with coloured-words (red, blue, yellow, and green) printed either in ink matching the colour denoted by the word or in a colour not matching that denoted by the word in a computer. Congruent and incongruent trials were presented with equal probabilities. Subjects were instructed to respond to the ink colour of the words by pressing one of four response keys with maximal speed and accuracy. We collected the measurements of: (1) omission rate (OR), (2) error rate (ER), (3) accuracy rate (AR); (4) correct RT^16,19,20^.

### Memory tests

Memory tests are two components of the Wechsler Memory Scale-Third Edition (WMS-III). We select the Faces subtest and the Logical memory subtest (LM). The Faces subtest includes: (1) immediate face memory (IFM); (2) face delayed memory (DFM); (3) Face memory percentage (FMP). The logical memory subtest includes: (1) immediate logical memory (ILM); (2) logical memory learning (LML); (3) delayed logical memory (DLM); (4) logical memory percentage (LMP); (5) logical memory recognition (LMR).

### Data collection

Data, including age, sex, body mass index, and daytime sleepiness (determined using the ESS and PSQI) were recorded. Arterial blood tests were performed within one week of the neurocognitive tasks. All the participants completed the psychomotor vigilance, flanker, and Stroop tasks, as well as the memory tests in a quiet, isolated room in the morning, without any interference. The questionnaires and tasks were completed at baseline within 1 week of the polysomnographic study.

### Statistical analysis

Independent -t tests were used to compare scores on the ESS, PSQI, psychomotor vigilance task (PVT), and flanker and Stroop tasks, and those on all of the memory tests. *P* values < 0.05 were considered statistically significant. All statistical analyses were performed using the statistical package SPSS for Windows (Version 14.0, SPSS; Chicago, IL).

## Results

### Demographic data

Of the 39 enrolled obese patients with OSA, 15 were hypercapnic (arterial blood PaCO_2_ ≧ 45 mmHg and pH ≧ 7.35), which consistent to diagnosis of obesity hypoventilation syndrome (OHS). Our study thus included 24 education-matched normocapnic subjects (OSA without diurnal hypoventilation). There was no difference between the two groups in age, BMI, or polysomnographic data, including AHI, ODI, and arousal index. There were only significant differences in PaCO_2_ (mean PaCO2, hypercapnic: 52.75 ± 8.40, normocapnic: 39.65 ± 3.58) between the two groups (Table 1).

**Table 1:** Demographic data.

Characteristics	PaCO_2_ ≧ 45 (N = 15)		PaCO_2_ < 45 (N = 24)	
	Mean (SD)		Mean (SD)	
Sex, Men (%):	66.67		91.67	
Age	51.87	(11.67)	48.54	(11.05)
BMI	38.18	(9.02)	34.88	(4.09)
PaCO2	52.75#	(8.40)	39.65	(3.58)
AHI	69.74	(25.11)	61.69	(25.93)
ODI	58.24	(28.55)	53.46	(24.20)
AI	35.57	(23.99)	37.08	(25.22)
ESS	11.86	(3.92)	12.71	(5.71)
PSQI	11.07	(3.71)	11.04	(4.94)

AHI: apnoea-hypopnoea index; AI: arousal index; BMI: body mass index; ESS: Epworth Sleep Scale; ODI: oxygen desaturation index; PSQI: Pittsburgh Sleep Quality Index; SD: standard deviation.

^#^P < 0.005.

### ESS and PSQI questionnaires

The mean ESS score was 11.86 ± 3.92 in the hypercapnic group and 12.71 ± 5.71 in the normocapnic group. The mean PSQI score was 11.07 ± 3.71 in hypercapnic patients and 11.04 ± 4.94 in normocapnic patients. There were no significant differences between the groups in ESS or PSQI scores (Table 1).

### Attention and Processing speed tasks

#### PVT

There was a significant increase in both, lapse rate and RT; and a decreased fast rate in the PVT in the hypercapnic group when compared to the normocapnic group (Table 2).

**Table 2:** Psychomotor vigilance test, Stroop task, flanker task and processing speed test.

Characteristics	PaCO_2_ ≧ 45 (N = 13)		PaCO_2_ < 45 (N = 24)	
	Mean (SD)		Mean (SD)	
**PVT**				
OR (%)	12.08	(14.72)	4.43	(5.49)
LR (%)	32.77*	(26.87)	8.03	(10.13)
HR (%)	87.87	(14.73)	95.57	(5.49)
FR (%)	67.18*	(26.87)	91.97	(10.13)
RT	350.49*	(38.26)	308.86	(48.26)
1/RT	2.70*	(0.65)	3.38	(0.58)
False RT	660.70	(243.94)	478.75	(310.04)
Fastest 10% RT	288.94*	(55.68)	250.47	(44.03)
Slowest 10% RT	627.65*	(287.88)	400.53	(106.54)
**Stroop task**				
OR (%)	5.59	(5.99)	2.92	(5.95)
ER (%)	5.53	(7.82)	6.6	(5.70)
AR (%)	88.88	(11.58)	91.31	(7.76)
Correct RT	905.27*	(185.60)	733.2	(157.19)
**Flanker task**				
OR (%)	2.37	(1.94)	1.25	(1.44)
ER (%)	12.22	(17.63)	5.27	(9.61)
AR (%)	85.41	(17.87)	93.47	(10.90)
Correct RT	451.89*	(79.57)	395.04	(69.60)
PS(processing speed)	54.67*	(22.90)	71.75	(16.84)

AR: accuracy rate; ER: error rate; FR: fast rate; OR: omission rate; RT: reaction time; SD: standard deviation.

*p < 0.05.

#### Processing speed (DSST)

Processing speed and all measures of logical memory were significantly worse in the hypercapnic patients with OSA than in the normocapnic patients with OSA (Table 2).

#### Executive tasks

Stroop task: No significant differences in the omission rate, error rate, or accuracy rate were observed in the congruent and incongruent Stroop tests. However, the correct RT was longer in the hypercapnic group (mean ± standard deviation [SD]: 905.27 ± 18.6 ms) than in normocapnic patients with OSA (mean ± SD: 733.2 ± 157.19 ms). The longer RTs were observed in both the congruent and incongruent tests in the Stroop task (Table 2).

#### Flanker task

In the flanker task, a significantly increased correct RT was observed in the hypercapnic patients (mean ± SD: 451.89 ± 79.57 ms) when compared to the normocapnic patients (mean ± SD: 395.04 ± 69.6 ms). The increased correct RTs were found in both, the congruent and the incongruent trials, in the flanker task. However, no differences in omission rate, error rate, or accuracy rate in the flanker test were found between the two groups (Table 2).

#### Memory tests

We assessed two memory parameters in our study: logical memory (immediate logical memory, logical memory learning, delayed logical memory, logical memory percentage, and logical memory recognition), and visual memory (immediate face memory, delayed face memory, and face memory percentage). There were no significant differences in the verbal memory between the two groups (Table 3).

**Table 3:** Memory test.

Characteristics	PaCO_2_ ≧ 45 (N = 13)		PaCO_2_ < 45 (N = 24)	
	Mean (SD)		Mean (SD)	
**ILM(**immediate logical memory)	30.87*	(12.72)	42.92	(13.09)
**LML(**logical memory learning)	17.73^#^	(10.00)	3.96	(1.90)
**DLM(**delayed logical memory)	2.67*	(1.88)	27.08	(8.84)
**LMP(**logical memory percentage**)**	74.35*	(26.43)	89.06	(15.93)
**LMR(**logical memory recognition)	22.07*	(4.13)	25.38	(3.19)
**IFM(**immediate face memory)	35.20	(4.13)	35.33	(4.20)
**DFM((**face delayed memory)	33.40	(3.68)	35.58	(4.37)
**FMP(**face memory percentage)	95.34	(8.35)	101.12	(10.07)

DFM: delayed face memory; DLM: delayed logical memory; FMP: face memory percentage; IFM: immediate face memory; ILM: immediate logical memory; LML: logical memory learning; LMP: logical memory percentage; LMR: logical memory recognition; SD: standard deviation.

*p < 0.05.

^#^p < 0.005.

Table 4 shows linear regression of each significant neurocognitive tasks and memory test with clinical and polysomnographic characteristics. LR, FR and RT was correlated to ODI, AHI and daytime PaCO2 levels. RT of Stroop task but not flanker task was correlated with daytime PaCO2 levels. In the other hands, most of the memory dysfunction(ILM, DLM, LMP) correlated with age and daytime PaCO2 levels. Processing process impairment was associated with age, arousal index and daytime PaCO2 levels.

**Table 4:** Linear regression model.

PVT				
variable	LR		FR	
	coefficients(β)	t	coefficients(β)	t
Age	0.133	0.79	−0.133	−0.79
ODI	1.118*	2.944	−1.118*	−2.944
AI	−0.024	−0.161	0.024	0.161
AHI	−0.841*	−2.178	0.841*	2.178
PaCO2	0.449*	2.943	−0.449*	−2.943
**variable**	**1/RT**		**RT**	
	**coefficients(β)**	**t**	**coefficients(β)**	**t**
Age	−0.146	−0.815	0.155	0.897
ODI	−0.829*	−2.043	0.90*	2.293
AI	−0.075	−0.46	0.077	0.487
AHI	0.882*	2.136	−1.079*	−2.703
PaCO2	−0.376*	−2.309	0.324*	2.056
**variable**	**Fastest 10% RT**		**Slowest 10% RT**	
	**coefficients(β)**	**t**	**coefficients(β)**	**t**
Age	0.254	1.438	0.093	0.51
ODI	1.037*	2.596	0.858*	2.072
AI	−0.057	−0.353	0.102	0.617
AHI	−1.093*	−2.688	−0.593	−1.408
PaCO2	0.170	1.057	0.357*	2.145
**Stroop&Flanker tasks**				
**variable**	**Stroop task Correct RT**		**Flanker task Correct RT**	
	**coefficients(β)**	**t**	**coefficients(β)**	**t**
Age	0.287	1.769	0.365	2.131*
ODI	0.514	1.399	0.344	0.889
AI	0.275	1.855	0.260	1.647
AHI	−0.488	−1.303	−0.432	−1.101
PaCO2	0.431*	2.916	0.314	2
**Memory**				
**variable**	**ILM**		**DLM**	
	**coefficients(β)**	**t**	**coefficients(β)**	**t**
Age	−0.399*	−2.432	−0.424*	−2.836
ODI	−0.080	−0.215	0.083	0.244
AI	−0.029	−0.194	0.032	0.234
AHI	−0.008	−0.021	−0.291	−0.844
PaCO2	−0.389*	−2.614	−0.458#	−3.368
	LMP		LMR	
variable	coefficients(β)	t	coefficients(β)	t
Age	−0.275	−1.717	−0.405*	−2.406
ODI	0.293	0.807	0.225	0.59
AI	0.161	1.098	0.057	0.37
AHI	−0.660	−1.782	−0.221	−0.567
PaCO2	−0.431*	−2.957	−0.284	−1.853
**Processing speed**				
**variable**	**coefficients(β)**	**t**		
Age	−0.469#	−4.411		
ODI	−0.244	−1.016		
AI	−0.340#	−3.504		
AHI	0.487	1.984		
PaCO2	−0.467#	−4.833		

DLM: delayed logical memory; FR: fast rate;; ILM: immediate logical memory; LML: logical memory learning; LMP: logical memory percentage; LMR: logical memory recognition; LR: lapse rate; PVT: Psychomotor vigilance test.

*p < 0.05.

^#^*p* < 0.005.

## Discussion

In the present study, obese patients with OSA and stable hypercapnia (OHS) had deficits in logical memory and attention, as well as increased correct RT in executive tasks when compared to those with OSA without diurnal hypoventilation. However, although the hypercapnic obese patients with OSA required longer to perform the executive and attention tasks, there were no differences in accuracy or error rate in the cognitive tasks. No differences in working memory or visual memory were found between hypercapnic and normocapnic patients with OSA. With the multiple regression, prolonged reaction time was correlated with intermittent nocturnal hypoxemia and daytime hypercapnia, as well as impaired momory was related with age and daytime hypercapnia.

Many studies have reported deficits in memory and cognitive ability in patients with OSA compared to individuals without OSA^5,9,21^. Most of these studies conclude that the memory or cognitive impairments were due to the pathophysiology of intermittent hypoxemia.

A few studies have addressed the importance of monitoring hypercapnia in the daytime or during sleep. Wang *et al*.^22^ have shown that hypercapnia may slow brain neural activity and lead to neurobehavioral impairments. In another brain imaging study, breathing 5% CO_2_ significantly suppressed all magnetic resonance imaging indices of functional connectivity^23^. In addition, animal studies have shown that hypercapnia leads to slower electroencephalography (EEG) activity^24,25^. In this study, we show that hypercapnia delayed the reaction time in cognitive function tests and was correlated with deficits in logical memory, but not verbal memory, in the obese patients with OSA. This finding highlights the importance of hypercapnia to cognitive and memory dysfunction in patients with OSA.

Previous studies have reported significant neurocognitive impairment in patients with OSA. Lee *et al*. have reported impairments in sustained attention on the PVT^26^. Furthermore, Tulek^27^ *et al*. have reported impairments in the executive function of the patients with OSA when compared to the normal subjects, in a neuroimaging study showing decreased activation in the anterior cingulate cortex associated with OSA^28^. In addition, cognitive impairments have been noted in patients with COPD, although the relationship among cognitive impairment, hypoxemia and hypercapnia is still debated^29^. In the present study, daytime hypercapnia was associated with increased RT in attention (PVT) and executive function (Stroop task and flanker task) tasks in patients with OSA.

In addition to attention and executive impairments, memory impairments have also been reported in the patients with OSA and COPD^21,29^. A majority of studies have reported impairments in the short-term verbal^5^ and visual memory, as well as the long-term semantic memory^30^ and procedural memory^31^ in the patients with OSA. Memory impairments have also been noted in COPD with underlying chronic hypoxia-hypercapnia pathology in animals^11^, although the effects of hypercapnia in human beings are not well-understood. In the present study, we demonstrated that hypercapnia impaired processing speed and logical memory, but not visual or working memory in obese patients with OSA.

On the other hand, ODI was not different between the groups, but hypercapnia was. Moreover, some studies reported an increase in the blood pressure during apnoea-hypopnea. This may be due to the exacerbation of nocturnal hypoxemia and hypercapnia as a result of apnoea-hypopnea. The severity of hemodynamic changes due to apnoea- hypopnea may be related to the duration of apnoea- hypopnea episodes, which can vary from 10 seconds to 1 minute, but may not be reflected in the AHI and ODI scores. Further, prolonged apnoea events may even lead to a decrease in the AHI and ODI index^32^. The mechanism and relationship between PSGdeoxygenation index and hypercapnia should be investigated in the future research.

Our study had some limitations. First, the hypercapnic obese OSA group had a small sample size. Second, we used a daytime blood test to define hypercapnia rather than nocturnal non-invasive blood carbon dioxide monitoring. Furthermore, the blood carbon dioxide levels were not determined during the neurocognitive tests. In addition, research including larger samples of patients with OSA should be considered for future studies.

In conclusion, not all the measured parameters in the neurocognitive tests were worse in OHS than OSA without diurnal hypoventilation. However, daytime stable hypercapnia prolonged the RT in the cognitive and attention function tests and led to deficits in logical memory function in obese patients with OSA without diurnal hypoventilation.

## References

[1] Young T (1993). The occurrence of sleep-disordered breathing among middle-aged adults. N. Engl. J. Med..

[2] The American Academy of Sleep Medicine Task Force (1999). Sleep-related breathing disorders in adults, recommendations for syndrome definition, measurement techniques in clinical research. Sleep.

[3] Stranks EK, Crowe SF (2016). The Cognitive Effects of Obstructive Sleep Apnea: An Updated Meta-analysis. Archives of Clinical Neuropsychology, 2016.

[4] Lal C, Strange C, Bachman D (2012). Neurocognitive impairment in obstructive sleep apnoea. Chest.

[5] Verstraeten E, Cluydts R, Pevernagie D, Hoffmann G (2004). Executive function in sleep apnoea: controlling for attentional capacity in assessing executive attention. Sleep.

[6] Twigg GL (2010). Obstructive sleep apnoea syndrome is associated with deficits in verbal but not visual memory. Am. J. Respir. Crit. Care Med..

[7] Jackson ML, Howardm MD, Barnesm M (2011). Cognition and daytime functioning in sleep-related breathing disorders. Prog. Brain Res..

[8] Bucks RS, Olaithe M, Eastwood P (2013). Neurocognitive function in obstructive sleep apnoea:A meta-review. (Respirology).

[9] Salorio CF, White DA, Piccirillo J, Duntley SP, Uhles ML (2002). Learning, memory, and executive control in individuals with obstructive sleep apnoea syndrome. J. Clin. Exp. Neuropsychol..

[10] Kheirandish L, Gozal D, Pequignot JM, Pequignot J, Row BW (2005). Intermittent hypoxia during development induces long-term alterations in spatial working memory, monoamines, and dendritic branching in rat frontal cortex. Pediatr. Res..

[11] Mitschelen M (2009). Basal and hypercapnia-altered cerebrovascular perfusion predict mild cognitive impairment in aging rodents. Neurosci..

[12] Andreou G, Vlachos F, Makanikas K (2014). Effects of chronic obstructive pulmonary disease and obstructive sleep apnoea on cognitive functions: evidence for a common nature. Sleep Disord..

[13] Olaithe M, Bucks RS, Hillman DR, Eastwood PR (2018). Cognitive deficits in obstructive sleep apnoea: Insights from a meta-review and comparison with deficits observed in COPD, insomnia, and sleep deprivation. Sleep Med Rev. 2018..

[14] Johns MW (1991). A new method for measuring daytime sleepiness: The Epworth sleepiness scale. Sleep.

[15] Buysse DJ, Reynolds CF, Monk TH, Berman SR, Kupfer DJ (1989). The Pittsburgh Sleep Quality Index (PSQI): A new instrument for psychiatric research and practice. Psychiatry Res..

[16] Shen, Y. C, Kung, S. C, Chang, E. T, Hong, Y. L, Wang, L. Y. The impact of obesity in cognitive and memory dysfunction in obstructive sleep apnea syndrome. *International journal of obesity*. **28**, 10.1038/s41366-018-0138-6 (2018). 10.1038/s41366-018-0138-6 29955085

[17] Dinges DF, Powell JW (1985). Microcomputer analyses of performance on a portable, simple visual RT task during sustained operations. Behav. Res. Methods Instrum. Comput..

[18] Eriksen BA, Eriksen CW (1974). Effects of noise letters upon identification of a target letter in a non-search task. Percept. Psychophys..

[19] Macleod CM (1991). Half a century of research on the Stroop effect: an integrative review. Psychol. Bull..

[20] Macleod CM, McDonald PA (2000). Interdimensional interference in the Stroop effect, uncovering the cognitive and neural anatomy of attention. Trends Cogn. Sci..

[21] Wallace A, Buck RS (2013). Memory and obstructive sleep apnoea: A meta-analysis. Sleep..

[22] Wang D, Thomas RJ, Tee BJ, Grunstein RR (2016). Hypercapnia is more important than hypoxia in the neuro-outcomes of sleep disordered breathing. J. Appl. Physiol..

[23] Xu F (2011). The influence of carbon dioxide on brain activity and metabolism in conscious humans. J. Cereb. Blood. Flow Metab..

[24] Forslid A, Ingvar M, Rosen I, Ingvar DH (1986). Carbon dioxide narcosis: influence of short-term high concentration carbon dioxide inhalation on EEG and cortical evoked responses in the rat. Acta Physiol. Scand..

[25] Zappe AC, Uludağ K, Oeltermann A, Uğurbil K, Logothetis NK (2008). The influence of moderate hypercapnia on neural activity in the anesthetized nonhuman primate. Cereb. Cortex.

[26] Lee IS (2011). The relation between psychomotor vigilance performance and quality of life in obstructive sleep apnoea syndrome. J. Clin. Sleep Med..

[27] Tulek B, Atalay NB, Kanat F, Suerdem M (2013). Attentional control is partially impaired in obstructive sleep apnoea syndrome. J. Sleep Res..

[28] Zhang X, Ma L, Li S, Wang Y, Wang L (2011). A functional MRI evaluation of frontal dysfunction in patients with severe obstructive sleep apnoea. Sleep Med..

[29] Dodd JW, Getov SV, Jones PW (2010). Cognitive function in COPD. Eur. Respir. J..

[30] Ferini-Strambi (2003). Cognitive dysfunction in patients with obstructive sleep apnoea (OSA): partial reversibility after continuous positive airway pressure (CPAP). Brain Res. Bull..

[31] Naegele B (2006). Which memory processes are affected in patients with obstructive sleep apnoea? An evaluation of 3 types of memory. Sleep.

[32] Wu H, Zhan X, Zhao M, Wei Y (2016). Mean apnoea-–hypopnea duration (but not apneanotapnea–hypopnea index) is associated with worse hypertension in patients with obstructive sleep apnoea. Medicine.

